# Health literacy and nutrition literacy among mother with preschool children: What factors are effective?

**DOI:** 10.1016/j.pmedr.2023.102323

**Published:** 2023-07-19

**Authors:** Farzane Ahmadi, Farzaneh Karamitanha

**Affiliations:** Faculty of Medicine, Zanjan University of Medical Sciences, Zanjan, Iran

**Keywords:** Health literacy, Nutrition literacy, Mother‘s literacy, Preschool children, Child health, Health promotion

## Abstract

Children's obesity behaviors are associated with parents' low health literacy. Nutrition Literacy (NL) is an essential component to enhancing nutrition quality, as well as health and well-being. The study's objective was to determine health literacy (HL) and NL among mothers with children preschool children (under 7 years old in Zanjan city, Iran).

Participants in this cross-sectional were 162 mothers with preschool children referred to Ayatollah Mousavi Hospital's pediatric clinic in Zanjan, Iran, during July and August 2022. The sample technique was convenient. In IBM SPSS 20 software, a two-step cluster analysis, the Chi-square\ Fisher exact, and Man-Whitney tests were used.

HL and NL had a mean ± SD of 71.26 ± 15.84 and 71.78 ± 11.86, respectively. The two-step cluster analysis identified two clusters, with mothers in cluster 1 (n = 141) having sufficient HL and NL and mothers in cluster 2 (n = 21) having insufficient HL and NL. Cluster 1 mothers were older, had a college degree, lived in a city, were employed, and had a better financial situation. They obtained health and nutrition information from the Internet, either alone or in conjunction with other sources.

Based on clustering analysis, it was observed that the low level of education, living in the village and being a housewife of the mothers, is related to the low level of HL and NL. By increasing the level of education of mothers, it is possible to help protect children from malnutrition or overfeeding in the future. Also, providing suitable job opportunities for mothers can help in this matter.

## Highlights

• A direct relationship was found between health literacy and nutrition literacy of mothers.
• For mothers with sufficient literacy, the main source of essential information was the Internet.
• The components of health literacy had greater importance in differentiating mothers into adequate and insufficient literacy.
• Mothers' employment and academic education increase mother's health and nutrition literacy.

## 1 Introduction

According to results of the Global Burden of Disease Study, nutritional risk factors are responsible for 22% of all deaths and 15% of all DALYs among adults. In addition, malnutrition, which includes insufficient nutrition, micronutrient deficiencies, and overweight, threatens the life, growth, and development of children and is caused by poor nutritional quality (Liu et al., 2021). Improper nutritional behavior is a risk factor for causing non-communicable diseases (Ashoori et al., 2021). A healthy diet is an important factor in the treatment and prevention of childhood obesity (Haszard et al., 2015). Parents play a central role in creating the suitable environment for children's physical activity and healthy nutrition. Children's nutritional behavior is greatly influenced by parents. Parents usually decide what food to prepare for the children and when and where they eat. Various studies have shown that parental style has an effect on children's obesity (Lindsay et al., 2018, Mais et al., 2017). Past studies have mentioned different reasons for the quality of the food we choose. Demographic, socio-economic, environmental, socio-cultural factors and nutrition knowledge and skills are among these factors (Ashoori et al., 2021). Education and literacy are important aspects of health promotion (Truman et al., 2020). Health Literacy (HL) is one of the important personal skills that enable people to control the determinants of health (Doustmohammadinan et al., 2021). Health literacy means accessing, understanding and using health information (Velardo, 2015). Limited HL is associated with worse health outcomes, and parents with limited HL have been shown to have more obesogenic behaviors (Taylor et al., 2019). In a study, it was reported that only 15% of parents had sufficient HL, which shows that most parents have problems in making health decisions, including choosing healthy food (Gibbs et al., 2016). Among the various influencing factors on nutritional behaviors, Nutrition Literacy (NL) has recently been proposed as a key factor in improving the quality of nutrition and health and well-being. NL is a concept that affects people's ability to receive food and nutritional information and read food labels and food safety precautions, as well as use healthy ways of cooking food and food recommendations to make healthy choices. Having healthy nutritional behaviors in children is very important and is essential for being physically healthy and learning optimally at school (Doustmohammadian et al., 2022). Recent studies have shown that NL is one of the key factors in having nutritional behaviors in children and adolescents (Doustmohammadinan et al., 2021). Also, NL as a part of HL can affect having healthier food choices (Truman et al., 2020). Various studies have shown that nutritional literacy is related to nutritional behaviors, and it has been observed that the increase in nutritional knowledge and literacy affects people's dietary changes (SaeidiFard et al., 2022). Considering that nutritional literacy is a form of health literacy (Velardo, 2015), the purpose of this study is to evaluate HL and NL among mothers with children under 7 years old in Zanjan city, Iran. Knowing the mothers' HL and NL levels, which is the person influencing children's healthy food choices, should be helpful for future interventions.

## 2 Methods

The cross-sectional study was carried out in Zanjan, Iran. Participants were mothers with preschool children (under 7 years old) referred to Ayatollah Mousavi Hospital's pediatric clinic, the only educational and therapeutic pediatric clinic in Zanjan, during July and August 2022. Zanjan is located in northwestern Iran at latitude 36.674339° and longitude 48.484467° east.

Based on the Fallah Morteza-Nejad et al., 2019, study, which indicated that the standard deviation (SD) of HL was 13.00, sample size was determined. It was calculated to be 162 using the Cochrane formula, $n\geq \frac{Z_{\frac{\alpha }{2}}^{2}\sigma^{2}}{d^{2}}$, with the assumptions of error sampling d = 2.00 and significance level 1-α=0.95. The sample technique was convenient. The number of mothers visiting the pediatric clinic was different according to the presence of different pediatricians. During the sampling period, days were selected to include mothers from different pediatricians. During the presence of the data collectors, the majority of mothers agreed to participate in the study. Mothers gave their first agreement after being told of the study's goals. The requirements for participation were being older than 18, having at least middle school, and having at least one kid less than seven years old. This study was approved by the Research Ethics Committees of Zanjan University of Medical Sciences, Zanjan, Iran (Approval Code: IR.ZUMS.REC.1401.090).

The demographic and socioeconomic information form, HL for Iranian Adults questionnaire, and the NL scale make up the three components of the data collection tool.

Demographic and socioeconomic variables include age, education level, weight (Kilograms) and height (Centimeters) to compute Body Mass Index (BMI, kg/m^2^), residence, job status, the number of children, underlying disease, the household income monthly (Toman), and the number of family members (the household size). To adjust the household income monthly (adjusted household income, AHI), it was divided by the household size. Then, based on quartiles, AHI was classified into 4 categories, from poor to rich. In addition, we asked mothers: how to get information related to health and nutrition?

The standard questionnaire HL for Iranian Adults was used to measure the HL. There are 33 items total, with 5 components (reading with 4 items, access to information with 6 items, understanding with 7 items, appraisal with 4 items, and decision making and behavior intention with 12 questions). This questionnaire used a Likert score with 5 possible options, (reading skills: quite hard = 1 to quite easy = 5), (4 other skills: never = 1 to always = 5). This questionnaire's validity and reliability were investigated by Montazeri et al., and Cronbach's alpha values varied from 0.72 to 0.89 (Tavousi et al., 2020). The individual item scores are added to calculate the component score. The sum of the five component scores is used to compute the total score. The component and total scores are converted to a scale of 0 to 100, and the higher the score, the greater HL. There are four different levels of HL: insufficient (≤50 points), not quite sufficient (50.1–66 points), sufficient (66.1–84 points), and excellent (≥84.1 points).

The third part was the Persian version of Evaluation Instrument of NL on Adults that was used to estimate nutrition status (Cesur et al., 2015) and that was standardized in Iran by (Hemati et al., 2018).The questionnaire has 35 items in 5 dimensions: general nutritional information (10 items), nutritional content understanding (6 items), food group determination (10 items), number of food units (3 items), and reading and understanding of food labels (6 items). The Kuder-Richardson Formula 20 yielded a value of 0.73, indicating internal consistency reliability (Cesur et al., 2015). There is only one right answer for each item. Items that were left unanswered or wrongly answered received 0 points, whereas each correct response received one point. Dimensions' and total scores were converted to a score out of 100. NL is classified as insufficient (≤24 points) or sufficient (>25 points).

The categorical variables were reported as frequency (percent) and continuous variables as mean ± SD or Median ± Interquartile Range (IQR). The Spearman correlation coefficient was used to assess the association between HL and NL and their components.

To identify similar subgroups (clusters) of mothers within the dataset, a two-step cluster analysis for five components of HL and NL was utilized. The reason for choosing cluster analysis was to answer the following questions:

1. Is there heterogeneity in HL and NL of mothers that can define different subgroups?
2. Which components of HL and NL play a more important role in the classification of HL and NL?
3. Which demographic and socioeconomic variables are the subgroups different from?

The two-step cluster analysis is a statistical approach that is more reliable and accurate than typical clustering algorithms such as k-means. The number of clusters was determined by Bayesian Information Criteria (BIC), with the optimal number of clusters being those with the lowest BIC. In additiona, the cohesion and separation Silhouette index was utilized to evaluate the clustering performance, which was classified as poor (less than0.2), fair (0.2–0.5), or good (>0.5) (Wendler and Gröttrup, 2016).

The predictor importance is a measure of the importance of a variable for cluster formation derived by a two-step algorithm that ranges from 0 to 1. The variables with low predictor importance (<0.4) had a little impact on the clusters formation. It was used to assess the significant HL and NL components in clustering. The components with the least predictor importance were removed, and the analysis was repeated until the Silhouette index exceeded 0.5 (good quality).

Finally, after determining clusters, the Chi-square\Fisher Exact test and Man-Whitney test were employed two recognized the discriminant demographic and socioeconomic characteristics among mothers' clusters. SPSS 20 was used for the analysis, and the significance level was set at 0.05.

## 3 Results

The mean ± SD 162 mothers were 31.96 ± 7.81 in the range of 18–48 years. The majority of them (77.2%) lived in urban area and had a college degree (46.9%). About 56% (91 mothers) of them had a BMI less than 25, with 6 mothers having a BMI less than 18.5. Only 8 people had underlying diseases, including: two hypothyroid, two rheumatoid arthritis, two diabetes, one valvular heart disease, and one lupus. Most of them were housewives with 2 children. 14.2% of mothers did not know how to get the information they need, and 41.9% used a combination of sources (Table 1).

**Table 1:** Descriptive of mothers' demographic and socioeconomic characteristics.

**Variable**	**Category**	**Frequency**	**Percent**
Education	Middle school	18	11.1
	High school	23	14.2
	Diploma	45	27.8
	College	76	46.9
Residence	Urban	125	77.2
	Rural	37	22.8
Job	Housewife	85	52.5
	Employed	77	47.5
BMI	<25	91	56.2
	≥ 25	71	43.8
Number of children	1	45	27.8
	2	77	47.5
	3 or more	40	24.7
Underlying Disease	Yes	8	4.9
	No	154	95.1
AHI	Poor(<1.20)	42	25.9
	2(1.20–1.67)	39	24.1
	3(1.67–2.33)	40	24.7
	Rich(2.33–8.33)	41	25.3
Source to get information related to the health and nutrition	Asking doctors and healthcare workers	9	5.6
	Internet	55	34.0
	Mass communication	7	4.3
	I don't know how to get the information I need	23	14.2
	Internet & Asking doctors and healthcare workers	10	6.2
	Mass communication & Asking doctors and healthcare workers	7	4.3
	Mass communication & Internet	20	12.3
	Internet & Mass communication & Asking doctors and healthcare workers	31	19.1

Appraisal and number of food units had the lowest levels of HL and NL, respectively. The component of *understanding and reading* and *understanding of food labels* had the best score in HL and NL, respectively. HL was categorized as insufficient with 15 (9.3%), not quite sufficient with 43 (26.5%), sufficient with 64 (39.5%), and excellent with 40 (24.7%). Also, NL was divided into three groups: insufficient, borderline, and sufficient, with 1 (0.6%), 53 (32.7%), and 108 (66.7%), respectively (Table 2).

**Table 2:** Mean ± SD, minimum, and maximum HL and NL components.

**Variable**	**Component**	**Mean ± SD**	**Minimum-Maximum**
HL	Reading	70.76 ± 20.68	19–100
	Access to information	70.47 ± 18.17	4–100
	Understanding	75.13 ± 19.16	7–100
	Appraisal	69.44 ± 18.77	25–100
	Decision making/behavioral intention	70.49 ± 16.16	31–100
	Total	71.26 ± 15.84	34–99

NL	General nutritional information	72.90 ± 20.24	10–100
	Nutritional content understanding	80.66 ± 19.39	16.67–100
	Food group determination	63.15 ± 17.95	10–90
	Number of food Units	54.94 ± 30.29	0–100
	Reading and understanding of food labels	83.85 ± 17.07	33.33–100
	Total	71.78 ± 11.86	31.43–88.57

Based on Table 3, there was no significant correlation between *food group determination* of NL with all components of HL, *the number of food units* of NL and *reading* of HL, and between *understanding nutrition information* of NL and *decision making/behavioral intention* of HL. However, there was a significant positive correlation between the other HL and NL components. The strongest and weakest significant associations was observed between *General nutritional information* (r = 0.412) and *the number of food units* of NL (r = 0.176) with *understanding* of HL, respectively.

**Table 3:** Spearman correlation coefficients between components of HL and NL.

**NL**	**HL**					
	Reading	Access to information	Understanding	Appraisal	Decision making/behavioral intention	Total
General nutritional information	r=0.340	r=0.317	r=0.412	r=0.318	r=0.264	r=0.381
	P<0.001	P<0.001	P<0.001	P<0.001	P=0.001	P<0.001
Nutritional content understanding	r=0.195	r=0.213	r=0.208	r=0.178	r=0.074	r=0.193
	P=0.013	P=0.006	P=0.008	P=0.023	P= 0.348	P=0.014
Food group determination	r=-0.114	r=-0.016	r=0.041	r=-0.033	r=-0.087	r=-0.049
	P=0.149	P=0.843	P=0.606	P=0.677	P=0.269	P=0.537
Number of food Units	r=0.207	r=0.070	r=0.176	r=0.218	r=0.187	r=0.186
	P=0.008	P=0.379	P=0.025	P=0.005	P=0.017	P=0.018
Reading and understanding of food labels	r=0.345	r=0.334	r=0.357	r=0.224	r= 0.225	r=0.343
	P<0.001	P<0.001	P<0.001	P=0.004	P= 0.004	P<0.001
Total	r= 0.275	r=0.274	r=0.396	r=0.274	r=0.188	r=0.321
	P<0.001	P<0.001	P<0.001	P< 0.001	P=0.017	P<0.001

**Fig. 1:**
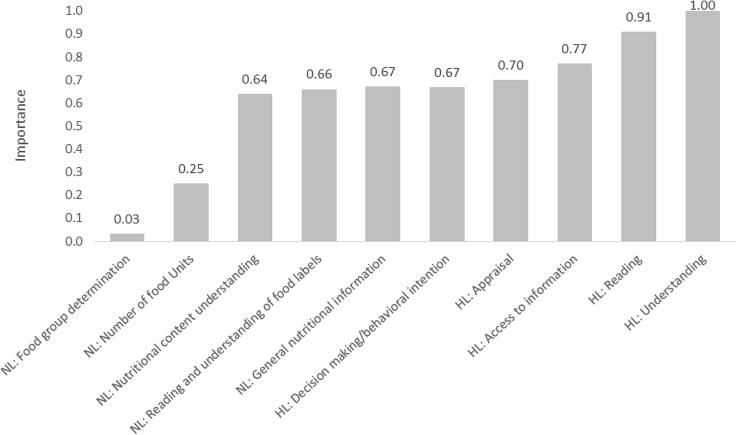
Value of HL and NL components predictor importance resulted from two-step cluster analysis.

According to the two-step cluster analysis, the optimal number of clusters was 2. Fig. 1 depicts the importance of 10 components of HL and NL from two-step cluster analysis. The model's Silhouette index was 0.46 (less than 0.5), so the clustering model had fair quality. *Food group determination* and *the number of food units* components of NL had the least importance value and did not meet the criteria of a predictor importance > 0.4 and lowered the Silhouette index. After deleting two these components, the algorithm found two groups again. In this case, the clustering model had good quality, with Silhouette index equals to 0.52. The two most important components were *nutritional content understanding* of NL and *understanding* of HL, with 1 and 0.95 importance values, respectively.

**Fig. 2:**
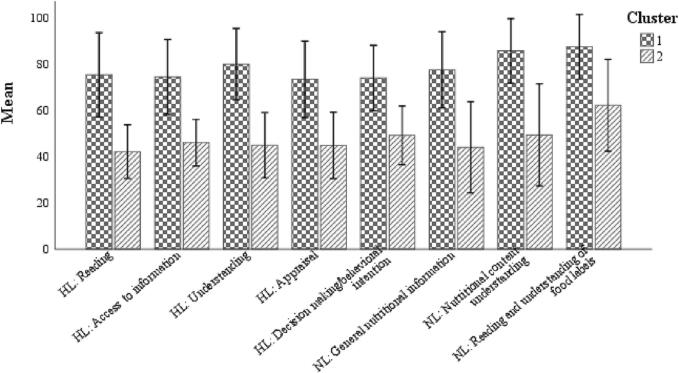
Mean ± SD components of HL and NL in two clusters resulted of two-step cluster analysis.

Fig. 2 represents the mean ± SD of the HL and NL components of the two clusters. Cluster 1 has higher HL and NL values than cluster 2. Fig. 2 and Table 4 show that the majority of mothers in the first cluster had a sufficient or excellent level of HL and sufficient level of NL (73.8% and 75.9%, respectively). However, in the second cluster, 100% of mothers had insufficient or not quite sufficient level of HL and 95.2% had borderline level of NL, respectively. As a result, cluster 1 was labeled as “sufficient literacy” and cluster 2 as “insufficient literacy”.

**Table 4:** Cluster comparison in terms of demographic and socioeconomic variables.

**Variable**	**Category**	**Cluster**		**Test statistics**	**P-value**
		**1:sufficient literacy n = 141**	**2:insufficient literacy n = 21**		
Total HL	Insufficient	1(0.7)	14(66.7)	101.78	<0.001§
	Not quite sufficient	36(25.5)	7(33.3)		
	Sufficient	64(45.4)	0(0)		
	Excellent	40(28.4)	0(0)		
Total NL	Insufficient	0(0)	1(4.8)	45.19	<0.001§
	Borderline	34(24.1)	19(90.5)		
	Sufficient	107(75.9)	1(4.8)		
Age	–	32.00 ± 13.00	27.00 ± 12.00	62272.00	<0.001^¶^
Education	Middle school	12(8.5)	6(28.6)	23.42	<0.001§
	High school	17(12.1)	6(28.6)		
	Diploma	36(25.5)	9(42.9)		
	College	76(53.9)	0(0)		
Residence	Urban	116(82.3)	9(42.9)	–	<0.001^£^
	Rural	25(17.7)	12(57.1)		
Job	Housewife	67(47.5)	18(85.7)	10.69	0.001¥
	Employed	74(52.5)	3(14.3)		
BMI	<25	82(58.2)	9(42.9)	1.74	0.187¥
	≥ 25	59(41.8)	12(57.1)		
Number of children	1	40(28.4)	5(23.8)	4.38	0.112¥
	2	70(49.6)	7(33.3)		
	3 or more	31(22.0)	9(42.9)		
Underlying Disease	Yes	7(5.0)	1(4.8)	–	>0.999^£^
	No	134(95.0)	20(95.2)		
AHI	Poor	29(20.6)	13(61.9)	16.41	0.001¥
	2	36(25.5)	3(14.3)		
	3	38(27.0)	2(9.5)		
	Rich	38(27.0)	3(14.3)		
Information Source of HL and NL	Asking doctors and healthcare workers	6(4.3)	3(14.3)	21.51	0.003§
	Internet	53(37.6)	2(9.5)		
	Mass communication	6(4.3)	1(4.8)		
	I don't know where to get the information I need	15(10.6)	8(38.1)		
	Internet & Asking doctors and healthcare workers	10(7.1)	0(0)		
	Mass communication & Asking doctors and healthcare workers	5(3.5)	2(9.5)		
	Radio & Internet	17(12.1)	3(14.3)		
	Internet & Mass communication & Asking doctors and healthcare workers	29(20.6)	2 (9.5)		

§ Chi-square test based on Monte Carlo simulation ^¶^: Mann-Whitney test, reported as median ± IQR.

¥ Chi-square test ^£^: Fisher Exact test.

Comparison results of two clusters based on study variables are reported in Table 4. There were significant differences between the two clusters in terms of age, education level, residence, job and AHI, such that mothers in cluster 1 (having sufficient literacy) were older, had a college degree, lived in an urban, were employed, and had better financial position. They also obtain knowledge on health and nutrition through the Internet, either alone or in conjunction with other sources. On the other hand, most mothers (38.1%) in the second cluster did not know how to obtain the necessary information. There was a relationship between levels of HL and NL and age, education level, residence, job, AHI, and source of HL and NL information.

## 4 Discussion

The present study was conducted with the aim of evaluating HL and NL of mothers with preschool children. The study results showed that most of the mothers participating in the study had a university education and more than half of them were housewives and city dwellers and had a normal body mass index. Most of the mothers participating in the study had 2 kids and their sources for obtaining nutritional and health information are the Internet. >65% of mothers had adequate NL and most of them had adequate HL.

Contrary to our study, Fallah Morteza-Nejad et al., 2019 study mentioned that the most common source of obtaining health information is asking doctors and health care workers. Various studies have been conducted in different contexts to investigate the level of HL and NL. The HL and NL level in some of these studies has been reported as adequate and sufficient (Fallah Morteza-Nejad et al., 2019, Habte et al., 2022, Moradzadeh et al., 2022). Various factors affect the HL and NL level. Many authors have mentioned that NL as a component of HL reflects the ability to access, interpret and use nutritional information (Velardo, 2015). Studies have shown that there is a relationship between parents' NL and children's food quality, income, parents' age, parents' education level. It has been seen that there is a relationship between education level and obesity in high-income countries such as the United States (Gibbs et al., 2016, Habte et al., 2022). It has also been observed that there is a positive relationship between the level of HL and the level of education (Habte et al., 2022). Moradzadeh et al., 2022, study also showed that there is a positive relationship between mother's job and socioeconomic status and HL level.

The highest score of the NL subgroup was related to *reading and understanding of food labels*, and *understanding* also had the highest score for HL. Based on the results of the analysis that was done to investigate the relationship between NL and HL, it was found that overall NL has a significant positive relationship with HL (r = 0.32).

Among the subgroups of HL, the highest positive correlation is related to the subgroup of *understanding* HL and *general nutritional information* and the lowest significant relationship between the sub-groups of NL and HL is related to the *number of food units* and *understanding*. *Food group determination* did not have a significant relationship with any of the subgroups of HL.

Using cluster analysis, it was determined that there were two groups of mothers based on HL and NL. In order to determine these two groups of mothers, two components, the *number of food units* and *food group determination* portions were less, and they were removed from the analysis during a two-stage analysis. Among the remaining components, the sub-groups of *nutritional content understanding* and *understanding* had the greatest contribution in differentiating mothers into two groups based on these two literacy levels. Based on this, it was seen that mothers with sufficient NL and HL have a higher age and education level, are employed, have a suitable income level, and live in the city. Most mothers with insufficient NL and HL had a diploma level of education. More than half of them lived in villages and most of them were housewives. The results of the study by KELEŞ ERTÜRK and ÖNAY DERİN, 2019, also showed a significant relationship in all NL scores with mother's occupation, monthly income, family type (nuclear, extended and broken) and mother's education level. Al Tell et al., 2023, also showed these cases in their study and mentioned that people with low socioeconomic and education levels and unemployed have less knowledge in the field of nutrition.

Sources of information for mothers with sufficient NL and HL were first the Internet, then the mass media, and finally asking doctors and health system employees. However, mothers with insufficient NL and HL did not know from which source to obtain the necessary information. This has also been seen in similar studies. It has been observed that people with high NL levels use more reliable sources for nutritional information, such as doctors and nurses, the Internet, and books, and are less likely to engage in inappropriate eating habits (Al Tell et al., 2023).

## 5 Conclusion

Adequate HL and NL are especially important for women. According to the results of clustering analysis, it was observed that the low level of education, living in the village and being a housewife of mothers, is related to the low level of health and nutritional literacy and there should be interventions in this Areas to improve the level of HL and NL in the communities. By increasing mothers' education levels, it is possible to help protect children from malnutrition or overfeeding in the future. Also, providing suitable job opportunities for mothers can help in this matter.

## Declaration of Competing Interest

The authors declare that they have no known competing financial interests or personal relationships that could have appeared to influence the work reported in this paper.

## Data Availability

Data will be made available on request.
